# Treatment outcomes of drug-resistant tuberculosis in the Netherlands, 2005–2015

**DOI:** 10.1186/s13756-019-0561-z

**Published:** 2019-07-12

**Authors:** Ivan S. Pradipta, Natasha van’t Boveneind-Vrubleuskaya, Onno W. Akkerman, Jan-Willem C. Alffenaar, Eelko Hak

**Affiliations:** 1 Groningen Research Institute of Pharmacy, Unit of Pharmaco-Therapy, - Epidemiology & - Economics (PTE2), University of Groningen, Antonius Deusinglaan 1, 9713 AV Groningen, the Netherlands; 2 Department of Pharmacology and Clinical Pharmacy, Faculty of Pharmacy, Universitas Padjadjaran, Jawa Barat, Indonesia; 3 Center of Excellence in Higher Education for Pharmaceutical Care Innovation, Universitas Padjadjaran, Jawa Barat, Indonesia; 4 Department of Clinical Pharmacy and Pharmacology, University Medical Centrum Groningen, Groningen, the Netherlands; 5 Department of Public Health Tuberculosis Control, Metropolitan Public Health Services , the Hague, the Netherlands; 6 Department of Pulmonary Diseases and Tuberculosis, University Medical Centre Groningen, University of Groningen, Groningen, the Netherlands; 7 Tuberculosis Centre Beatrixoord, University Medical Centre Groningen, University of Groningen, Haren, the Netherlands; 8 Faculty of Medicine and Health, School of Pharmacy, University of Sydney, Sydney, Australia; 9 Westmead Hospital, Sydney, Australia

**Keywords:** Tuberculosis, Risk factors, Predictors, Unsuccessful treatment, Mortality, Epidemiology

## Abstract

**Background:**

Since in low incidence TB countries population migration and complex treatment of drug-resistant tuberculosis (DR-TB) patients are major issues, we aimed to analyse patient risk factors associated with the incidence of poor outcome of TB treatment among DR-TB patients in the Netherlands.

**Methods:**

This retrospective cohort study included adult patients with confirmed DR-TB treated from 2005 to 2015. We obtained data from a nationwide exhaustive registry of tuberculosis patients in the Netherlands. Predictors for unsuccessful TB treatment (defaulted and failed treatment) and TB-associated mortality were analysed using multivariate logistic regression.

**Results:**

Among 10,303 registered TB patients, 545 patients with DR-TB were analysed. Six types of DR-TB were identified from the included patients, i.e. isoniazid mono- or poly-resistance (68%); rifampicin mono- or poly-resistance (3.1%); pyrazinamide mono-resistance (8.3%); ethambutol mono-resistance (0.1%); multidrug-resistance (18.9%); and extensively drug-resistance (0.7%). The majority of patients were foreign-born (86%) and newly diagnosed TB (89%) patients. The cumulative incidence of unsuccessful treatment and mortality were 5 and 1%, respectively. Among all DR-TB cases, patients with Multi Drug-Resistant Tuberculosis (MDR-TB) (OR 4.43; 95%CI 1.70–11.60) were more likely to have unsuccessful treatment, while miliary and central nervous system TB (OR 15.60; 95%CI 2.18–111.52) may also be predictors for TB mortality. Additionally, patients with substance abuse and homelessness tend to have unsuccessful treatment.

**Conclusions:**

In recent years, we identified a low incidence of DR-TB as well as the poor outcome of DR-TB treatment. The majority of cases were primary drug-resistant and foreign-born. To further improve treatment outcome, special attention should be given to the high-risk DR-TB patients.

**Electronic supplementary material:**

The online version of this article (10.1186/s13756-019-0561-z) contains supplementary material, which is available to authorized users.

## Background

Drug-resistant tuberculosis (DR-TB), infection with a strain of *M. tuberculosis* (*M. tb*) that is resistant to one or more of the first-line anti-tuberculosis drug, is an ongoing global threat. DR-TB can be classified into mono-, rifampicin-, poly-, multidrug- and extensive drug- resistance. The World Health Organization (WHO) globally recorded 160,684 cases of multidrug-resistant/ rifampicin-resistant tuberculosis (MDR/RR-TB) in 2017 [1]. However, treatment success remains low at 55% globally [1] and the cost of treating Multi- or Extensively Drug-resistant Tuberculosis (M/XDR-TB) is up to 25 times higher than the cost of drug-susceptible tuberculosis [2].

Although in the WHO European region the fastest decline in incidence and mortality rate of TB has been reported since 2010 [3], DR-TB remains out of control. One-third of notified MDR-TB cases identified globally are people who live in the WHO European Region, and additional resistance commonly exists with MDR-TB in this region [4]. Furthermore, XDR-TB shows an increasing trend. Among 91.3% second-line Drug Susceptibility Test (DST), 18.6% of pulmonary MDR-TB cases had XDR-TB in 2017 [5]. A recent study showed different rates of treatment success, treatment failure and death of MDR-TB patients in 16 European countries [6]. The problem is more complex as travel and migration of people have been identified as a risk factor of TB burden in the European countries [7, 8]. This can lead to the transmission of DR-TB from high to low incidence TB countries which in increasingly being reported from some European countries [9, 10].

The Netherlands is one of the low incidence TB countries [8]. The government has formulated a national tuberculosis control plan that set 1 case/100.000 people as a target for TB elimination by 2035 [11]. Previously published studies reported highly successful treatment of MDR-TB in the Netherlands from 1985 to 2009 [12, 13]. However, these studies neither analysed all types of drug-resistant TB nor identified predictors for poor outcome of TB treatment. Hence, updated data are required to describe the current situation, evaluate current programmes and identify potential interventions to improve treatment outcomes of the overall DR-TB types in the Netherlands as well as to achieve the national target. Since mono- or poly-resistant TB can potentially develop into the poor outcome of TB treatment and a further level of resistance, we therefore aimed to determine the prevalence of different types of DR-TB cases and its characteristics of the not-evaluated patient for the treatment outcome over the recent years from 2005 to 2015 in the Netherlands. Additionally, we also examined the incidence and predictors for poor outcome of TB treatment among all DR-TB patients and the subgroup of MDR-TB patients.

## Methods

### Study design and setting

We conducted a retrospective cohort study using a database from the Netherlands Tuberculosis Registry (NTR) covering the period from January 1, 2005 to December 31, 2015. De-identified data were obtained from the NTR on January 23, 2018. The NTR is an exhaustive nationwide database for tuberculosis disease in the Netherlands managed by the Dutch National Institute for Public Health and the Environment (RIVM). Real-time surveillance data are routinely collected by RIVM in close collaboration with the TB control department of the Municipal Public Health Services (MPHS) and the Royal Netherlands Tuberculosis Association (KNCV). NTR data collection occurs throughout the TB diagnostic and treatment period, and the information is entered by the physician or nurse into an electronic report via the Online Registration System for Infectious Diseases in the Infectious Diseases Surveillance Information System (OSIRIS) after the diagnosis is made. The data were validated by KNCV and MPHS for the completeness and consistency through an interactive process [14]. MPHS received reminders when the data entered in OSIRIS were incomplete and online system enables MPHS to correct and add the information.

### Study patients

In the present study, adult DR-TB patients in the Netherlands were our population of interest. We included adult patients 18 years and older who were diagnosed with tuberculosis disease during the period 2005–2015, caused by *M. tb* pathogen proven to be resistant to at least one of the first-line antituberculosis drugs. A phenotypical confirmation test has been used as a standard test in the Netherlands between 2005 and 2007. However, a combination test, i.e., phenotypic test (Bactec MGIT 960 system) and genotypic test (Genotype MTBDR plus assay or Line Probe Assay (LPA)), have been applied since 2007. Drug susceptibility testing (DST) was conducted to determine resistance to first-line anti-tuberculosis drugs. If the resistance had been confirmed, the DST was extended to the second-line drugs, except for isoniazid, pyrazinamide and ethambutol mono-resistance. We retrospectively followed-up up to 24 months for patients identified as DR-TB. The observation was started from the time the diagnosis of DR-TB was made to the outcome of TB treatment was reported. Patients who had not started treatment and had an unknown treatment outcome were excluded from the analysis for the incidence and patient risk factors for poor outcome of TB treatment. Moreover, patients who had an unknown treatment outcome were included for further analysis of a not-evaluated patient outcome.

### Potential predictors and definitions

Potential predictors were identified at baseline of TB diagnosis, and were selected from a previous meta-analytical study [15], input from TB practitioners and information from the NTR database. Five categories of potential predictors were analysed in this study, including socio-demographic information (age, gender, country of birth, and domicile area), current TB diagnosis (type of pulmonary TB diagnosis, initial TB location, country of the TB diagnosis, and type of drug resistance), history of TB disease and treatment (BCG vaccination, previously diagnosed TB and treated latent TB infection / LTBI), risk groups (TB contacts, immigrants, asylum seekers, homeless individuals, health care workers, travellers from high endemic area, prisoners, alcohol dependence, and drugs dependence), and comorbidities (diabetes, malignancies, HIV, renal insufficiency/on dialysis, and organ transplantation). Operational definitions of the variables and terminology followed the definitions stated in the OSIRIS and WHO guideline [14, 16] (See Additional file 1: Table S1).

### Study outcomes

We defined unsuccessful TB treatment and TB associated mortality as the primary outcome for the predictors of poor outcome treatment. Unsuccessful treatment was a combination of defaulted and failed treatments, while TB associated mortality was mortality due to tuberculosis disease that was defined by a doctor who treated the patient. Defaulted treatment was defined as such if one of the following four conditions was met: 1) an interruption of TB treatment, that was not decided by the clinician, for two or more consecutive months, 2) an uncompleted 6-month treatment in a 9-month period, 3) an uncompleted 9-month treatment in a 12-month period, and 4) treatments where patients took less than 80% of their medication [14, 16]. Failed treatments were defined as having a TB-positive sputum smear or culture on the fifth month or later after treatment initiation [14, 16]. In case of RR/M/XDR-TB, treatment failure was defined if one of the following five conditions was met: 1) lack of conversion by the end of the intensive phase, 2) bacteriological reversion in the continuation phase after conversion to negative, 3) evidence of additional acquired resistance to fluoroquinolone or second-line injectable drugs 4) adverse drug reactions, or 5) a TB-positive sputum smear or culture were defined after 12 month or later from the initial TB treatment [14, 16]. As a secondary outcome, we studied the characteristic of patients who were not evaluated for the treatment outcome. The patient who started the treatment but were unknown for the treatment outcome, e.g., in transferred out cases, were defined as not-evaluated patients.

### Data analysis

We used descriptive analysis to describe characteristics of the study patients, trends of DR-TB cases and incidence of poor treatment outcomes during the study period. The cumulative incidence was used to express incidence for the poor outcome by dividing the number of cases of poor outcomes of TB treatment (unsuccessful treatment or TB-associated mortality) by the number of patients diagnosed with DR-TB. A univariate analysis was conducted for each of the potential predictors and outcomes. We used the chi-square test or the Fisher’s exact test (when expected cell size was < 5) for the categorical data in the univariate analysis. Potential predictors that had a *p*-value ≤0.15 were included in the multivariate analysis. The logistic regression analysis with a backward step elimination based on a *p*-value > 0.05 and entry method were used for the multivariate analysis. We used a complete case analysis in the multivariate analysis considering the low percentage of the missing data from the variables analysed. We identified 1 (0.2%) missing values for gender, 2 (0.4%) missing values for country of birth, 53 (9.7%) missing values for newly diagnosed TB, and 58 (10.6%) missing values for previous LTBI treatment. Furthermore, some potential predictors were not included in the analysis due to a high level of missing values, i.e. presence of HIV (51%) and BCG vaccination (51%). To quantify the level of the association between predictors and the outcome, an odds ratio (OR) with 95% confidence interval (95%CI) was calculated. Calibration values of the final model were assessed by the Hosmer-Lemeshow test. Statistical Package for the Social Science version 23 was used for the statistical analysis in this study, and we followed the STROBE statement for reporting the study results [17].

## Results

**Fig. 1:**
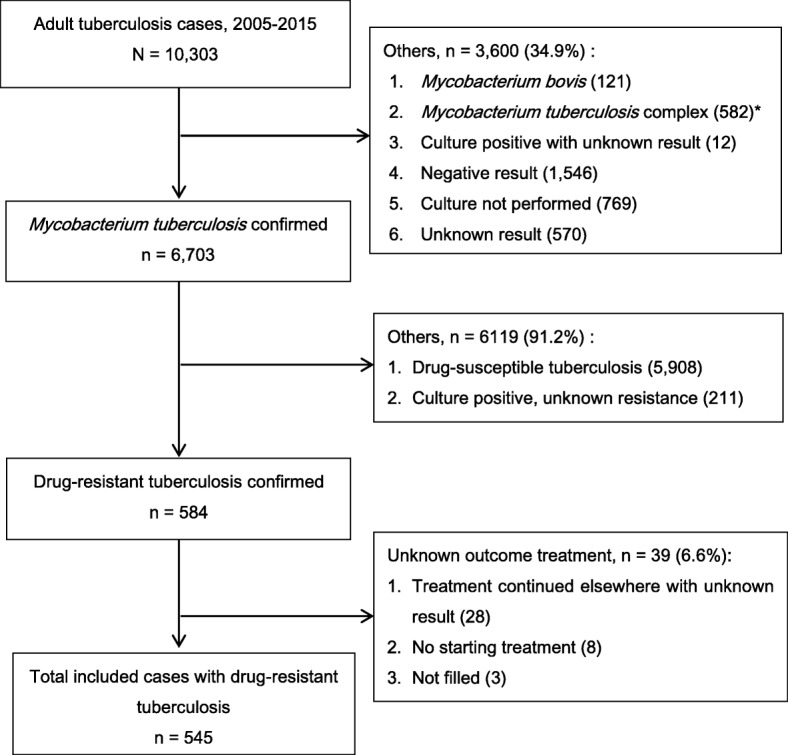
Flow diagram of the included patients. *The proportion of drug-resistant *M. tb* complex was 8.2% (49/582); The proportion of *M. tb* complex with the known type of strain was 1.2% (7/582)

Out of 10,303 adult TB cases identified during the study period, we included 545 (5.3%) DR-TB cases that fulfilled criteria of the study (See flowchart in Fig. 1). During the same period, the prevalence of MDR-TB was 1% (n/*N* = 103/10,303).

**Fig. 2:**
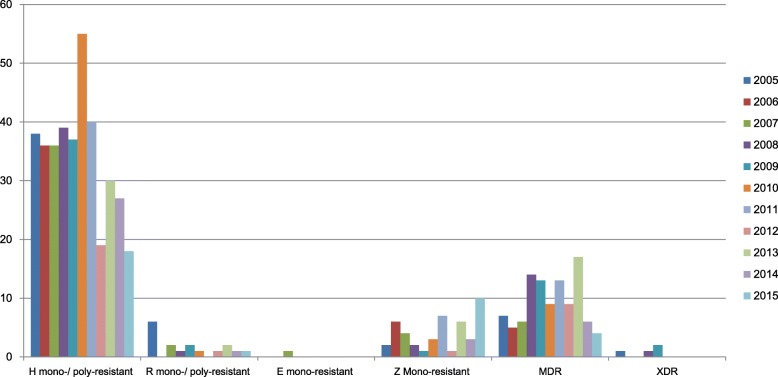
The number of drug-resistant cases in the Netherlands from 2005-2015. Notes: H, isoniazid; R, rifampicin; E, ethambutol; Z, pyrazinamide; MDR, multidrug-resistant; XDR, extensively drug-resistant

We identified that the highest proportion of DR-TB during the study period existed of isoniazid mono- or poly-resistant TB cases (375 cases), while the highest number of patients diagnosed with all type of DR-TB was in 2010 (68 cases). As the second highest proportion of DR-TBs, MDR-TB was identified in each of the years, with 103 diagnosed cases during the study period. However, some types of DR-TB, such as rifampicin mono- or poly-resistant strains, ethambutol mono-resistant and XDR-TB, were not consistently found every year during the study period. Overall, there was a declining trend of DR-TB cases during the study period, from 54 cases (2005) to 33 cases (2015) (Fig. 2).

With regard to the patient risk factors, DR-TB patients were slightly more often in the male (54%), rural domicile (66%), and pulmonary TB diagnosis (52%) group, while most cases were newly diagnosed with TB (88%), foreign-born (86%), isoniazid or rifampicin mono−/poly-resistant TB (72%), with TB diagnosis in the Netherlands (98%) and aged between 25 and 64 years old (74%) (Table 1).

**Table 1:** Characteristics of patients (*n* = 545)

No	Characteristics	Frequency (%)
1	Socio-demographic	
	Male^a^	295 (54.1)
	Age (years):	
	18–24	106 (19.4)
	25–64	404 (74.1)
	65+	35 (6.4)
	Country of birth^a^:	
	Somalia	109 (20)
	The Netherlands	74 (13.6)
	Morocco	41 (7.5)
	Indonesia	28 (5.1)
	Others	293 (53.8)
	Rural domicile^b^	359 (65.9)
2	Current TB diagnosis	
	Pulmonary diagnosis:	
	ETB	191 (35)
	PTB	283 (51.9)
	ETB + PTB	71 (13)
	Type of TB location:	
	Lungs	333 (61.1)
	Miliary and central nervous system TB	13 (2.4)
	Respiratory tract	38 (7)
	Intestinal tract	15 (2.8)
	Bone and joint	28 (5.1)
	Urogenital tract	9 (1.7)
	Other organ	109 (20)
	Diagnosed by doctors abroad	11 (2)
3	History of TB disease & treatment	
	Previously diagnosed TB^a^	59 (10.8)
	Previously treated LTBI^a^	24 (4.4)
4	The risk group of TB	
	TB contacts	29 (5.3)
	Immigrants	68 (12.5)
	Asylum seekers	87 (16)
	Illegal immigrants	14 (2.6)
	Homeless individual	15 (2.8)
	Alcohol dependence	8 (1.5)
	Substance abuse	18 (3.3)
	Health care workers	4 (0.7)
	Travelers from/in endemic areas for more than three month	19 (3.5)
	Prisoners	12 (2.2)
5	Comorbidities	
	Diabetes	18 (3.3)
	Malignancy	11 (2)
	Insufficient renal function or on dialysis	5 (0.9)
	Organ transplantation	2 (0.4)

Information: ^a^missing value: Gender 1 (0.2%), Country of birth 2 (0.4%), Newly diagnosed TB 53 (9.7%), previous LTBI treatment 58 (10.6%); ^b^Urban domicile: Amsterdam, Rotterdam, the Hague and Utrecht; TB, tuberculosis; ETB, extra-pulmonary tuberculosis; PTB, pulmonary tuberculosis; LTBI, latent tuberculosis infection

As described in Fig. 1, we identified 28 patients with unknown treatment outcome. We observed that previously diagnosed TB patients, illegal immigrants, travelers from/in endemic areas and prisoners were more likely to be not evaluated for their treatment outcome (*p* < 0.05) (Additional file 1: Table S2).

### Treatment outcomes and its predictors among the overall drug-resistant tuberculosis patients

We observed that there was no failed treatment outcome in DR-TB patients. The treatment outcomes among the overall DR-TB patients (*N* = 545) were cured treatment (*n* = 44), completed treatment (*n* = 463), defaulted treatment (*n* = 25), TB-associated mortality (*n* = 6), and non-TB-associated mortality (*n* = 7). Therefore, the cumulative incidence of unsuccessful treatment and death were 5 and 1%, respectively. In the univariate analysis, several variables such as being male, having MDR-TB, homelessness, alcohol dependence, and substance abuse were significantly associated with unsuccessful treatment (*p* < 0.05). Furthermore, we included those predictors together with potential predictors that have a *p* ≤ 0.15 in the multivariate analysis. Finally, we identified three significant predictors (p < 0.05) for the unsuccessful treatment of TB in the multivariate analysis, i.e., having MDR-TB (OR 4.43; 95% CI 1.70–11.60), homelessness (OR 9.10; 95% CI 2.32–35.74), and substance abuse (OR 6.66; 95% CI 1.72–25.82). Performance of the final model was acceptable, with a Hosmer-Lemeshow test *p*-value of 0.88 (Table 2).

**Table 2:** Predictors for the unsuccessful treatment of tuberculosis among drug-resistant tuberculosis patients (*n* = 545)

No	Predictors	Unsuccessful treatment		Univariate analysis		Multivariate analysis*	
		No (*n* = 520)	Yes (*n* = 25)	Odds ratio (95% CI)	*p*-value	Odds ratio (95% CI)	*p*-value
1	Socio-demographic						
	Male^a^	275 (53)	20 (80)	3.55 (1.31–9.60)	0.008	2.30 (0.79–6.69)	0.13
	Age (years):				0.88	Not included	–
	18–24	101 (19.4)	5 (20)	Ref.	–	–	–
	25–64	385 (74)	19 (76)	0.99 (0.36–2.74)	–	–	–
	65+	34 (6.5)	1 (4)	0.59 (0.06–5.27)	–	–	–
	Foreign-born patients^a^	447 (86.3)	22 (88)	1.12 (0.34–3.99)	0.81	Not included	–
	Urban domicile^b^	174 (33.5)	12 (48)	0.55 (0.24–1.22)	0.13	1.85 (0.74–4.63)	0.19
2	Current TB diagnosis						
	Pulmonary diagnosis:				0.07		0.39
	ETB	188 (36.2)	3 (12)	Ref.	–	Ref.	–
	PTB	265 (51)	18 (72)	4.26 (1.24–14.66)	–	2.44 (0.66–9.05)	–
	ETB + PTB	67 (12.9)	4 (16)	3.74 (0.82–17.15)	–	2.50 (0.49–12.66)	–
	Type of TB location:				0.20	Not included	–
	Lungs	313 (60.2)	20 (80)	2.48 (0.92–6.71)	–	–	–
	Miliary and central nervous system	13 (2.5)	0 (0)	n/a	–	–	–
	Others	194 (37.3)	5 (20)	Ref.	–	–	–
	Diagnosed by doctors abroad	11 (2.1)	0 (0)	n/a	–	–	–
	Type of resistance:				0.03		0.05
	Isoniazid mono−/poly-resistant	365 (70.2)	10 (40)	Ref.	–	Ref.	–
	Rifampicin mono−/poly-resistant	16 (3.1)	1 (4)	2.28 (0.28–18.9)	–	1.68 (0.19–15.22)	–
	Pyrazinamide/ ethambutol mono-resistant	43 (8.3)	3 (12)	2.54 (0.68–9.61)	–	2.96 (0.73–12.07)	–
	MDR-TB	92 (17.7)	11 (44)	4.36 (1.80–10.59)	–	4.43 (1.70–11.60)	–
	XDR-TB	4 (0.8)	0 (0)	n/a	–	n/a	–
3	History of TB disease & treatment						
	Previously diagnosed with TB^a^	55 (11.7)	4 (17.4)	1.59 (0.52–4.83)	0.51	Not included	–
	Previously treated LTBI^a^	24 (5.2)	0 (0)	n/a	0.62	Not included	–
4	Risk group of TB						
	TB contacts	27 (5.2)	2 (8)	1.59 (0.36–7.09)	0.54	Not included	–
	Immigrants	65 (12.5)	3 (12)	0.96 (0.28–3.28)	0.94	Not included	–
	Asylum seekers	81 (15.6)	6 (24)	1.71 (0.66–4.42)	0.26	Not included	–
	Illegal residence persons	13 (2.5)	1 (4)	1.63 (0.20–12.94)	0.49	Not included	–
	Homeless individuals	9 (1.7)	6 (24)	17.93 (5.79–55.50)	0.000	9.10 (2.32–35.74)	0.002
	Alcohol dependence	5 (1)	3 (12)	14.05 (3.15–62.54)	0.004	4.35 (0.60–31.31)	0.14
	Substance abuse	12 (2.3)	6 (24)	13.37 (4.53–39.43)	0.000	6.66 (1.72–25.82)	0.006
	Health care workers	4 (0.8)	0 (0)	n/a	0.66	Not included	–
	Travellers from/in endemic areas for more than 3 month	19 (3.7)	0(0)	n/a	0.33	Not included	–
	Prisoners	10 (1.9)	2 (8)	4.44 (0.92–21.42)	0.10	0.43 (0.05–3.94)	–
5	Comorbidities						
	Diabetes	17 (3.3)	1 (4)	1.23 (0.16–9.65)	0.58	Not included	–
	Malignancy	11 (2.1)	0 (0)	n/a	0.46	Not included	–
	Insufficient renal function or on dialysis	5 (1)	0 (0)	n/a	0.62	Not included	–
	Organ transplantation	2 (0.4)	0 (0)	n/a	0.76	Not included	–

Information: *The cases were analysed using backward elimination method in the multivariate analysis; Hosmer & Lemeshow test 0.88; n/a: not applicable due to small number of event; Ref.: Reference; Not included: the predictor was not included due to *p*-value > 0.15 in the univariate analysis; ^a^missing value: Gender 1 (0.2%), Country of birth 2 (0.4%), Newly diagnosed TB 53 (9.7%), previous LTBI treatment 58 (10.6%); ^b^Urban domicile: Amsterdam, Rotterdam, The Hague and Utrecht; TB, tuberculosis; ETB, extra-pulmonary tuberculosis; PTB, pulmonary tuberculosis; MDR-TB, multidrug-resistant tuberculosis; XDR-TB, extensively drug-resistant tuberculosis; LTBI, latent tuberculosis infection; CI, confidence interval

In a univariate analysis, we found miliary and central nervous system (CNS) TB (OR 14.96; 95% CI 2.47–90.52) as a potential predictor for the mortality outcome. Our final model in the multivariate analysis showed that miliary and CNS TB (OR 15.60; 95%CI 2.18–111.52) were more prone to having death as an outcome than any other TB site adjusted by variables of age and illegal immigrant. Our final model demonstrated an acceptable calibration with a Hosmer-Lemeshow test p-value of 0.85 (Table 3).

**Table 3:** Predictors for mortality outcomes due to tuberculosis among drug-resistant tuberculosis patients (*n* = 545)

No	Predictors	TB-associated mortality		Univariate analysis		Multivariate analysis*	
		No (*n* = 539; %)	Yes (*n* = 6; %)	Odds ratio (95% CI)	*p*-value	Odds ratio (95% CI)	*p*-value
1	Socio-demographic						
	Male^a^	292 (54.3)	3 (50)	0.84 (0.17–4.21)	0.83	Not included	–
	Age (years):				0.07		0.05
	18–24	105 (19.5)	1 (16.7)	Ref.	–	Ref.	–
	25–64	401 (74.4)	3 (50)	0.78 (0.08–7.63)	–	0.65 (0.06–6.91)	–
	65+	33 (6.1)	2 (33.3)	6.36 (0.56–72.44)	–	8.24 (0.63–107. 05)	–
	Foreign-born patients^a^	464 (86.4)	5 (83.3)	0.78 (0.09–6.83)	0.58	Not included	–
	Urban domicile^b^	183 (34)	3 (50)	1.95 (0.39–9.73)	0.42	Not included	–
2	Current TB diagnosis						
	Pulmonary diagnosis:				0.23	Not included	–
	ETB	191 (35.4)	0 (0)	n/a	–	–	–
	PTB	280 (51.9)	3 (50)	0.24 (0.05–1.23)	–	–	–
	ETB + PTB	68 (12.6)	3 (50)	Ref.	–	–	–
	Type of TB location:				0.013		0.024
	Lungs	329 (61)	4 (66.7)	Ref.	–	Ref.	–
	Miliary and central nervous system	11 (2)	2 (33.3)	14.96 (2.47–90.52)	–	15.60 (2.18–111.52)	–
	Others	199 (36.9)	0 (0)	n/a	–	n/a	–
	Diagnosed by doctors abroad	11 (2)	0 (0)	0	0.72	Not included	–
	Type of resistance:				0.97	Not included	–
	Isoniazid mono−/poly-resistant	371 (68.8)	4 (66.7)	Ref.	–	–	–
	Rifampicin mono−/poly-resistant	17 (3.2)	0 (0)	n/a	–	–	–
	Pyrazinamide/ ethambutol mono-resistant	45 (8.3)	1 (16.7)	2.06 (0.23–18.85)	–	–	–
	MDR-TB	102 (18.9)	1 (16.7)	0.91 (0.10–8.23)	–	–	–
	XDR-TB	4 (0.7)	0 (0)	n/a	–	–	–
**3**	History of TB disease & treatment						
	Previously diagnosed with TB^a^	59 (12.1)	0 (0)	n/a	0.41	Not included	–
	Previously treated LTBI^a^	24 (5)	0 (0)	n/a	0.61	Not included	–
4	The risk group of TB						
	TB contacts	29 (5.4)	0 (0)	0	0.56	Not included	–
	Immigrants	67 (12.4)	1 (16.7)	1.41 (0.16–12.24)	0.55	Not included	–
	Asylum seekers	86 (16)	1 (16.7)	1.05 (0.12–9.13)	0.96	Not included	–
	Illegal residence persons	13 (2.4)	1 (16.7)	8.09 (0.88–74.24)	0.15	8.87 (0.71–111.40)	0.09
	Homeless individual	15 (2.8)	0 (0)	n/a	0.68	Not included	–
	Alcohol dependence	8 (1.5)	0 (0)	n/a	0.76	Not included	–
	Substance abuse	18 (3.3)	0 (0)	n/a	0.65	Not included	–
	Health care workers	4 (0.7)	0 (0)	n/a	0.83	Not included	–
	Travellers from/in endemic areas for more than 3 month	19 (3.5)	0 (0)	n/a	0.64	Not included	–
	Prisoners	12 (2.2)	0 (0)	n/a	0.71	Not included	–
5	Comorbidities						
	Diabetes	17 (3.2)	1 (16.7)	6.14 (0.68–55.46)	0.18	Not included	–
	Malignancy	11 (2)	0 (0)	n/a	0.72	Not included	–
	Insufficient renal function or on dialysis	5 (0.9)	0 (0)	n/a	0.81	Not included	–
	Organ transplantation	2 (0.4)	0 (0)	n/a	0.88	Not included	–

Information: * The cases were analysed using entry method in the multivariate analysis; Hosmer & Lemeshow test 0.85; n/a: not applicable due to small number of event; Ref.: Reference; Not included: the predictor was not included due to *p*-value > 0.15 in the univariate analysis; ^a^missing value: Gender 1 (0.2%), Country of birth 2 (0.4%), Newly diagnosed TB 53 (9.7%), previous LTBI treatment 58 (10.6%); ^b^Urban domicile: Amsterdam, Rotterdam, The Hague and Utrecht; TB, tuberculosis; ETB, extra-pulmonary tuberculosis; PTB, pulmonary tuberculosis; MDR-TB, multidrug-resistant tuberculosis; XDR-TB, extensively drug-resistant tuberculosis; LTBI, latent tuberculosis infection; CI, confidence interval

### Treatment outcomes and its predictors among the multidrug-resistant tuberculosis patients

Since MDR-TB was a risk factor for poor outcome treatment among all DR-TB patients, we attempted to gain more insight about the predictors of treatment outcome in the subgroup of MDR-TB patients. We observed that among the 103 MDR-TB cases, most cases were from the group of foreign-born patients, followed by those living in rural domiciles, having lung-TB, newly diagnosed with TB without any previous TB treatment and identified as DR-TB-positive in the Netherlands. Figure 2 reveals that there has been a fluctuating trend in the number of MDR-TB cases from 2005 to 2015. Treatment outcomes of the MDR-TB patients (*N* = 103) were cured treatment (*n* = 4), completed treatment (*n* = 85), defaulted treatment (*n* = 11), TB associated mortality (n = 1), and non-TB associated mortality (*n* = 2). Overall, the cumulative poor TB treatment outcome incidence (a combination of unsuccessful treatment and death due to tuberculosis) was 12%. The significant differences (*p* < 0.05) for the poor TB treatment outcome were found in patients with male gender, homelessness, and substance abuse in the univariate analysis. In the final model, male gender (OR 9.80; 95%CI 1.18–81.68) and substance abuse (OR 7.50; 95%CI 1.07–52.37) were identified as independent predictors for poor TB treatment outcomes in MDR-TB cases. A Hosmer-Lemeshow test was shown on *p*-value of 1 (Table 4).

**Table 4:** Predictors of poor outcomes of TB treatment among multidrug-resistant tuberculosis patients (*n* = 103)

No	Predictors	Non-Poor outcome (*n* = 91; %)	Poor outcome^b^ (*n* = 12; %)	Univariate analysis		Multivariate analysis*	
				Odds ratio (95% CI)	*p*-value	Odds ratio (95% CI)	*p-*value
1	Socio-demographic						
	Male	42 (46.2)	11 (91.7)	12.83 (1.59–103.57)	0.003	9.80 (1.18–81.68)	0.035
	Age (years):				0.42	Not included	–
	18–24	26 (28.6)	2 (16.7)	Ref.	–	–	–
	25–64	63 (69.2)	9 (75)	1.86 (0.38–9.19)	–	–	–
	65+	2 (2.2)	1 (8.3)	6.50 (0.39–106.71)	–	–	–
	Foreign-born patients^d^	89 (97.8)	12 (100)	n/a	0.99	Not included	–
	Urban domicile^c^	21 (23.1)	5 (41.7)	0.42 (0.12–1.46)	0.17	Not included	–
2	Current TB diagnosis						
	Pulmonary diagnosis:				0.88	Not included	–
	ETB	21 (23.1)	2 (16.7)	Ref.	–	–	–
	PTB	55 (60.4)	8 (66.7)	1.53 (0.30–7.79)	–	–	–
	ETB + PTB	15 (16.5)	2 (16.7)	1.40 (0.17–11.08)	–	–	–
	Type of TB location:				0.71	Not included	–
	Lungs	65 (71.4)	8 (66.7)	0.95 (0.23–3.86)	–	–	–
	Miliary and central nervous system	3 (3.3)	1 (8.3)	2.56 (0.19–33.16)	–	–	–
	Others	23 (25.3)	3 (25)	Ref.	–	–	–
	Diagnosed by a doctor abroad	5 (5.5)	0 (0)	n/a	0.41	Not included	–
3	History of TB disease & treatment						
	Previously diagnosed with TB^a^	18 (22.5)	4 (33.3)	1.72 (0.47–6.38)	0.47	Not included	–
	Previously LTBI treatment^a^	5 (6.4)	0 (0)	n/a	0.38	Not included	–
4	The risk group of TB						
	TB contacts	4 (4.4)	1 (8.3)	1.98 (0.20–19.32)	0.47	Not included	–
	Immigrants	20 (22)	1 (8.2)	0.32 (0.04–2.65)	0.45	Not included	–
	Asylum seekers	27 (29.7)	4 (33.3)	1.18 (0.33–4.27)	0.75	Not included	–
	Illegal residence persons	5 (5.5)	0 (0)	n/a	0.41	Not included	–
	Homeless individual	2 (2.2)	2 (16.7)	8.9 (1.13–70.26)	0.02	2.15 (0.19–24.28)	0.54
	Alcohol dependence	0 (0)	1 (8.3)	n/a	0.12	n/a	–
	Substance abuse	2 (2.2)	3 (25)	14.83 (2.18–100.78)	0.01	7.50 (1.07–52.37)	0.04
	Health care workers	1 (1.1)	0 (0)	n/a	0.72	Not included	–
	Travellers from/in endemic areas for more than 3 month	5 (5.5)	0 (0)	n/a	0.41	Not included	–
	Prisoners	4 (4.4)	0 (0)	n/a	0.46	Not included	–
5	Comorbidities						
	Diabetes	3 (3.3)	1 (8.3)	2.67 (0.26–27.92)	0.39	Not included	–
	Malignancy	2 (2.2)	0 (0)	n/a	0.60	n/a	–
	Insufficient renal function or undergoing dialysis	0 (0)	0 (0)	n/a	n/a	n/a	–
	Organ transplantation	0 (0)	0 (0)	n/a	n/a	n/a	–

Information: * The cases were analysed using backward step elimination method in the multivariate analysis; The Hosmer and Lemeshow test: 1.00; Ref.: Reference; Not included: the predictor was not included due to *p*-value > 0.15 in the univariate analysis; ^a^missing data: previously diagnosed TB: 11 (10.7%), Previously treated LTBI: 14 (13.6%). LTBI: Latent Tuberculosis Infection. ^b^Poor outcome of treatment is a combination of unsuccessful treatment and death outcome due to tuberculosis; ^c^Urban domicile: Amsterdam, Rotterdam, The Hague and Utrecht; n/a: not applicable due to small number of event; ^d^Foreign-born countries: Somalia 25 (24.3%), Georgia 8 (7.8%), Russia 6 (5.8%), India 5 (49%), Others 57 (55.33%)

## Discussion

Our study demonstrated that the overall prevalence and poor outcomes of DR-TB cases among adults in the Netherlands were relatively low. Most DR-TB cases were foreign-born, newly diagnosed TB and isoniazid mono−/poly-resistant TB patients. Though the numbers were low, we identified that MDR-TB, homelessness, and substance abuse were statistically significant predictors for unsuccessful treatment, while miliary and CNS-TB were analysed as predictors for TB-associated mortality among overall DR-TB cases. Additionally, we noted that patients with male gender and substance abuse were more likely to have a poor outcome after MDR-TB treatment. Among all DR-TB cases, we found that previously diagnosed TB patients, illegal immigrants, travelers from/in endemic areas and prisoners were more likely not to be evaluated for their treatment outcome, which indicates potential risk of poor outcome treatment.

Our study showed that the Netherlands has a low prevalence of DR-TB and poor DR-TB treatment outcomes. Several studies described that the prevalence of DR-TB and MDR-TB across the 27 European Union (EU) and European Economic Area (EEA) countries were 10 and 2%, respectively [18], while our data demonstrated that the Netherlands has a 5.3% prevalence of DR-TB and a 1% prevalence of MDR-TB. In case of MDR-TB, the treatment success rate in the Netherlands was 88%, which is higher than the globally reported rates (46–58%) [3] and the 27 EU/EEA countries (48%) [18].

Our study determined that homelessness and substance abuse are risk factors for having an unsuccessful TB treatment outcome in overall DR-TB patients. Homeless patients are faced with several problems, such as unstable accommodation, lack of infection awareness, difficulties of accessing healthcare services, stigmatization, problems with access to proper nutrition and suffering from comorbidities [19]. Those problems can lead to increasing discontinuation rate and non-adherence to the medication. A published review stated that drug users are associated with vulnerable TB condition, such as homelessness and HIV status [20]. It can be argued that homeless patients are a susceptible group to have poor TB treatment outcomes. Although due to low numbers of outcomes, we observed statistically significant associations. However, the precision of the estimates was low, especially for the factors homelessness and substance abuse.

CNS and miliary TB should be a concern in the management of TB as their mortality risk was the highest of all TB forms. This finding was supported by a study in Denmark [21] that showed that CNS-TB was a factor strongly associated with mortality in TB patients. Another study reported that CNS-TB was frequently accompanied with miliary TB [22]. The multifaceted problems in the management of CNS-TB relate to delays in clinical recognition, diagnosis, treatment and drug penetration in cerebrospinal fluid, have been determined as the main issues to improve successful treatment [23].

As expected, although isoniazid mono−/poly-resistant TB was presented in the majority of cases in this study, MDR-TB cases tend to have more frequently the unsuccessful treatment outcome. Additional use of moxifloxacin in first-line TB regimen may give a positive effect for the outcome of isoniazid mono−/poly-resistant TB patients. A meta-analytical study that included data from the Netherlands supported that addition of a fluoroquinolone to 6 months or more of first-line regimen was associated with significantly greater treatment success [24]. On the other hand, the complexity of the MDR-TB treatment regimen that uses a combination of first- and second-line drugs based on susceptibility testing can result in unsuccessful treatment. The treatment is longer, less effective and less tolerable than standard treatments, and involves injectable drugs as well. Hence, adverse events can occur among MDR-TB patient and are a factor in the decision to discontinued treatment.

We also found that males and substance abuse are associated with poor MDR-TB treatment outcomes. The finding of an association between gender and tuberculosis treatment outcomes remains a contested debate. Several studies have reported that there is no association between gender and treatment outcomes among DR-TB patients [25–27]. In contrast, studies in Nigeria [28] and Taiwan [29] found that male gender is associated with poorer tuberculosis treatment outcomes, while a review explained an opposite statement [30]. The disparity of the result can be explained by differences in social, cultural, economic and clinical factors between patients and geographical area. Financial dependence, cultural inequality and greater fear of the stigmatization make it more difficult for women to access qualified medical care in some areas [31, 32]. On the other hand, gender-specific social role makes men to have more social contact in other areas, thereby increasing the risk of TB exposure [33]. Furthermore, clinical aspects can also play a role in the treatment outcome. A study in Nigeria [28] showed that male patients were older, while a study in Taiwan [29] described that males were more likely to smoke, have COPD, malignancy, cirrhosis, low body weight, pleural effusion or hemoptysis. In our data, the prevalence of the poor outcome in MDR-TB was higher in males (10.68%) than females (0.97%). We found that substance abuse was the only one characteristic that associated with poor outcome in the male group, while there was no characteristic associated with poor outcome in the female group (see Additional file 1: Table S3). Although substance abuse was indicated as a factor that affected the poor outcome in the different gender, a further study that considers social, cultural, economic and clinical aspects is required to obtain a comprehensive picture across geographical areas.

The present study indicated that most DR-TB patients were foreign-born, with primary drug-resistant *M. tb*. This finding can be explained by the fact that the most DR-TB patients had a newly diagnosed TB Since the Netherlands has a low TB prevalence, it seems that immigration and activation of latent TB were essential factors of DR-TB cases in the Netherlands.

Several potential limitations in our study need to be mentioned. First, some potential predictors such as HIV status, treatment delay, history of BCG vaccine, level of education, the income of patients, and patients’ beliefs, could not be analysed due to the unavailability of the data for a large number of patients. Second, since the data were collected from a national database, we relied on administrative input without any direct investigation. Third, the low incidence of the study outcomes (unsuccessful treatment and death) led to potential overestimations and a wide confidence interval around the odd ratios in some associations between predictors and the outcomes. The inaccuracy of point estimate may exist in the association between gender and poor treatment outcome among MDR-TB patients. It is due to the uncommon incidence of poor treatment outcome in the female group. However, we identified that the probability of poor treatment outcome was significantly higher in the male (91.7%) than female group (8.3%). Additionally, a factor that was associated with poor treatment outcome in the MDR-TB group, i.e., substance abuse, was significantly dominated by male patients (Additional file 1: Table. S3). These reasons seem to suggest that males are more likely to have poor treatment outcome among MDR-TB patients. Fourth, analysis of the appropriateness of medication cannot be performed due to lack of detailed treatment history and regimen in the database. However, we believe that integrating documentation and data collection of TB information, supported with integrated information technology and a referral system of healthcare services in the Netherlands, will minimize potential bias and results can be generalized to the Dutch population. Importantly the information may also be useful for low-incidence TB countries in general.

A high success rate for MDR-TB treatment in the Netherlands was constantly reported from the previous studies [12, 13] to the present study. Integrated systems and collaboration between all stakeholders may be the key to this success. Municipal Public Health Services (MPHSs) have an important role in controlling TB in the Netherlands. Twenty-five MPHSs, staffed by public health TB control officers, physicians, nurses, and administrative staff, are spread widely across the Netherlands [34]. They have the responsibility to diagnose, treat and monitor TB and LTBI patients for TB control. Suspected TB patients from the general practice or at-risk groups, such as immigrants, asylum seekers, and prisoners will have a TB examination in MPHSs to identify TB cases. A dedicated hospital TB coordinator in the Netherlands manages TB cases in the hospital setting. To optimize treatment adherence, TB nurses in MPHSs have been trained as treatment supporters in order to monitor drug adherence during the treatment period. Two special hospitals for TB, called modern TB centres, are available for long-term admissions, socially problematic cases and clinically complex patients, such as TB meningitis or M- and XDR-TB patients [35]. If a contagious TB patient refuses treatment and poses a risk to the general population, the patient can be compulsorily isolated according to the Dutch Public Health Act. TB centre Beatrixoord is designated by the Dutch government for compulsory isolation. Moreover, pharmacokinetics/pharmacodynamics modeling has been used for therapeutic drug monitoring (TDM) in the MDR-TB treatment for years [36]. Since the treatment can be up to 24-month treatment, the TDM supported for shortening the regimen due to low drug exposure as well as improve safety and efficacy of the drugs [37].

However, to optimize treatment outcome among DR-TB patients, special attention should be given to patients with MDR-TB, homelessness, substance abuse, as well as miliary and CNS-TB. Admission of these patients to a modern TB centre may be an option to intensify the treatment and monitoring of these high-risk patients. It can also prevent further development of drug resistance and transmission of tuberculosis in the community [35]. The treatment management for these patients should not only focus on medical support but also on social support. Treatment should not only be seen from the perspective of delivery to the patients but should also be seen from a comprehensive care perspective that should consider the patient’s ability to take medicine, to make a right life choice, and the treatment should support their circumstances to ensure an adherence to the treatment and an improvement in the quality of life [20, 35].

## Conclusions

We observed a low incidence of poor tuberculosis treatment outcomes among DR-TB patients. The majority of DR-TB cases were foreign-born patients with a newly diagnosed TB. To avoid unsuccessful treatment amongst DR-TB patients in the Netherlands; special attention should be given to patients with MDR-TB, homelessness, and substance abuse. Furthermore, miliary and CNS TB treated in general hospitals should be monitored carefully and/or treated together with TB specialists, or admitted to a modern TB center, to prevent premature mortality due to TB. We also identified that patients with male gender and substance abuse were more likely to have poor MDR-TB treatment outcomes. Close monitoring should be given to DR-TB patients with previous TB diagnosis, illegal status, traveling status from/in endemic areas and prisoner status to decrease the number of not-evaluated patient outcome. Further studies are required to identify critical factors for poor TB treatment outcomes, particularly in identified high-risk groups.

## Additional file

Additional file 1: **Table S1.** The operational definition of study. **Table S2.** Characteristics of not-evaluated patients (*n* = 573). **Table S3.** Poor outcome of TB treatment between males and females among MDR-TB patients (*N* = 103). (PDF 391 kb)

## Data Availability

The datasets generated and/or analysed during the current study are not publicly available but are available from the corresponding author on reasonable request.

## Abbreviations

BCG: Bacillus Calmette–Guérin
CI: Confidence interval
CNS TB: Central Nervous System Tuberculosis
DR-TB: Drug-resistant tuberculosis
DST: Drug susceptibility test
EEA: European Economic Area
EU: European Union
HIV: Human Immunodeficiency Viruses
KNCV: Koninklijke Nederlandse Centrale Vereniging tot bestrijding der Tuberculose / The Royal Netherlands Tuberculosis Association
LTBI: Latent Tuberculosis Infection
*M. tb*: *Mycobacterium tuberculosis*
M/XDR-TB: Multi- or Extensively Drug-resistant Tuberculosis
MDR/RR-TB: Multidrug-resistant/ rifampicin resistant tuberculosis
MDR-TB: Multidrug-resistant tuberculosis
MPHS: Municipal Public Health Services
NTR: The Netherlands Tuberculosis Registry
OR: Odds ratio
OSIRIS: Online Registration System for Infectious Diseases in the Infectious Diseases Surveillance Information System
RIVM: Rijksinstituut voor Volksgezondheid en Milieu / the Dutch National Institute for Public Health and the Environment
STROBE: Strengthening The Reporting of OBservational Studies in Epidemiology
TB: Tuberculosis
TDM: Therapeutic drug monitoring
WHO: World Health Organization
XDR-TB: Extensively Drug-resistant Tuberculosis
