# Supplementary material for: Treatment outcomes of drug-resistant tuberculosis in the Netherlands, 2005–2015
Source: Antimicrob Resist Infect Control. 2019 Jul 12;8:115. doi: 10.1186/s13756-019-0561-z (PMC6626402; doi:10.1186/s13756-019-0561-z)
Supplement: Supplementary file 1 — Table S1. The operational definition of study. Table S2. Characteristics of not-evaluated patients (n = 573). Table S3. Poor outcome of TB treatment between males and females among MDR-TB patients (N = 103). (PDF 391 kb) [file 13756_2019_561_MOESM1_ESM.pdf]

Supplementary material

Title : Treatment outcomes of drug-resistant tuberculosis in the Netherlands, 2005-2015

Authors: Ivan S. Pradipta, Natasha van't Boveneind-Vrubleuskaya, Onno W. Akkerman, Jan-Willem C. Alffenaar, Eelko Hak

Table S1. The operational definition of study

No	Variables	Operational definition
Predictors		
1	Age	Age when the current diagnosis of TB was made
2	Pulmonary diagnosis	Type of pulmonary TB defined by a medical doctor based on an ICD code. It was divided into three categories: pulmonary TB (PTB), extra-pulmonary TB (ETB) and the combination of PTB and ETB.
3	Type of TB location	Type of localization of TB based on an ICD-9 defined by a medical doctor. It was divided into lung tuberculosis, central nervous system (CNS) and miliary TB, and other tuberculosis (respiratory tract, intestinal, urogenital, bone, joint and others)
4	Diagnosed by a doctor abroad	Patients who were diagnosed with TB outside the Netherlands and continue their treatment in the Netherlands
5	Previously diagnosed with TB	Patients who were previously diagnosed with TB based on clinical findings and or documentation.
6	Previously undergoing LTBI treatment	Patients who were previously treated for Latent Tuberculosis Infection (LTBI) based on clinical findings and or documentation
7	TB contacts	Patients who have been identified as having had contact with a TB patient.

8	Immigrants	Patients with a legal residence status other than a tourist or refugee or asylum seeker.
9	Asylum seekers	Patients who left their home country as political refugees and are seeking asylum in another country.
10	Illegal	Patients without legal residence status in the Netherlands at the time of diagnosis, regardless of the length of stay in the Netherlands.
11	Homelessness	Patients who have not had a fixed residence or regularly sleep on the street and/or use marginal temporary accommodation.
12	Health care workers	Patients who work as health care providers
13	Travelers from the high endemic area more than three month	Patients who have traveled from or to the high endemic area for more than three months
14	Prisoners	Patients who were staying in prison at the time of the diagnosis, including patients who were screened in prison, but the diagnosis was made after discharge from the prison.
15	Alcohol dependence	Patients who have problematic alcohol consumption at the time of diagnosis. Problematic alcohol is related to a drinking pattern that leads to physical complaints and/or psychological or social problems. The amount of alcohol consumed was not considered for defining the status of an alcoholic.
16	Substance abuse	Patients who have regularly used drugs, including narcotics, methadone and cocaine, which has led to some degree of social disorder.
17	Comorbidities	Patients who have a disease or compelling indication with TB defined by medical doctors. This was divided into diabetes mellitus, malignancy, insufficient renal function/on dialysis and organ transplantation.
18	Drug-resistant TB (DR-TB)	Patients who have resistance to at least one of the first-line anti-TB drugs. They were divided into sixth categories: 1) isoniazid mono-/ poly-resistant TB; 2) rifampicin mono-/ poly-resistant TB; 3) ethambutol mono-resistant TB; 4) pyrazinamide mono-resistant TB; 5) multidrug-resistant TB (MDR-TB; 6) extensively drug-resistant TB (XDR-TB).

19	Mono-resistant tuberculosis	Resistance to one of the first-line anti-TB drug only
20	Poly-resistant tuberculosis	Resistance to more than one first-line anti-TB drug (other than both isoniazid and rifampicin)
21	Isoniazid mono-/ poly-resistant tuberculosis	a resistant *M.tb* strain to isoniazid alone; OR poly resistant *M.tb* strain to isoniazid with one or more first-line anti-tuberculosis drugs other than rifampicin
22	Rifampicin mono-/poly-resistant tuberculosis	a resistant M. Tb strain to rifampicin alone; OR poly-resistant M. Tb strain to rifampicin with one or more first-line anti-tuberculosis drugs other than isoniazid
23	Multidrug-resistant tuberculosis	a *M.tb* strain that is resistant to at least both isoniazid and rifampicin.
24	Extensively drug-resistant tuberculosis	a MDR-TB in addition to resistance to any of the fluoroquinolones (levofloxacin or moxifloxacin) and at least one of the three injectable second-line drugs (amikacin, capreomycin or kanamycin)
Outcomes		
25	Unsuccessful treatment	A combination of defaulted and failed treatments.
26	Death	Patients who die due to tuberculosis disease, as defined by a doctor.
27	Not-evaluated	Patients who started the treatment but were unknown for the treatment outcome, including transferred out cases.
28	Poor TB treatment outcome	A combination of the unsuccessful TB treatment and death due to tuberculosis disease.

Table S2. Characteristics of not-evaluated patients (n= 573)

No	Characteristics	Not-evaluated patient		Odds ratio (95%CI)	p-value
		No (n=545)	Yes (n=28)		
1	Socio-demographic				
	Male**	295 (54.1)	20 (71.4)	2.04 (0.91-4.55)	0.07
	Age (years):				0.11
	18-24	106 (19.4)	4 (14.3)	Ref.	
	25-64	404 (74.1)	24 (85.7)	1.57 (0.54-4.64)	
	65+	35 (6.4)	0 (0)	n/a	
	Country of birth*:				0.02*
	The Netherlands	74 (13.6)	0 (0)	n/a	
	Somalia	109 (20)	2 (7.1)	0.22 (0.52-0.96)	
	Morocco	41 (7.5)	1 (3.6)	0.29 (0.04-2.26)	
	Indonesia	28 (5.1)	1 (3.6)	0.44 (0.06-3.35)	
	Others	293 (53.8)	24 (85.7)	Ref.	
	Urban domicile‡	186 (34.1)	11 (39.3)	1.24 (0.59-2.59)	0.58
2	Current TB diagnosis				
	Pulmonary diagnosis:				0.16
	PTB	283 (51.9)	19 (67.9)	Ref.	
	ETB	191 (35)	5 (17.9)	0.39 (0.14-1.06)	
	ETB + PTB	71 (13)	4 (14.3)	0.84 (0.28-2.54)	
	Type of TB location:				0.43
	Lungs	333 (61.1)	22 (78.6)	Ref.	
	Respiratory tract	38 (7)	1 (3.6)	0.39 (0.05-3.04)	
	Miliary and central nervous system	13 (2.4)	1 (3.6)	1.16 (0.15-9.31)	
	Intestinal tract	15 (2.8)	1 (3.6)	1.01 (0.13-7.80)	
	Bone and joint	28 (5.1)	1 (3.6)	0.54 (0.07-4.16)	
	Urogenital tract	9 (1.7)	0 (0)	n/a	
	Others	109 (20)	2 (7.1)	0.28 (0.06-1.20)	
	Diagnosed by doctors abroad	11 (2)	0 (0)	n/a	0.45
	Type of resistance:				0.97
	Isoniazid mono-/poly-resistant	375 (68.8)	20 (71.4)	Ref.	

	Rifampicin mono-/poly-resistant	17 (3.1)	0 (0)	n/a	
	Pyrazinamide/ ethambutol mono-resistant	46 (8.5)	0 (0)	n/a	
	MDR-TB	103 (18.9)	8 (28.6)	1.46 (0.62-3.40)	
	XDR-TB	4 (0.7)	0 (0)	n/a	
3	History of TB disease & treatment				
	Previously diagnosed with TB**	59 (12)	7 (31.8)	3.43 (1.34-8.75)	0.015*
	Previously treated LTBI**	24 (4.9)	0 (0)	n/a	0.99
4	The risk group of TB				
	TB contacts	29 (5.3)	0 (0)	n/a	0.39
	Immigrants	68 (12.5)	4 (14.3)	1.17 (0.39-3.47)	0.77
	Asylum seekers	87 (16)	3 (10.7)	0.63 (0.19-2.14)	0.60
	Illegal residence persons	14 (2.6)	4 (14.3)	6.32 (1.94-20.66)	0.009*
	Homeless individuals	15 (2.8)	2 (7.1)	2.72 (0.59-12.52)	0.19
	Alcohol dependence	8 (1.5)	0 (0)	n/a	0.99
	Substance abuse	18 (3.3)	2 (7.1)	2.25 (0.50-10.23)	0.26
	Health care workers	4 (0.7)	0 (0)	n/a	0.99
	Travellers from/in endemic areas for more than 3 month	19 (3.5)	5 (17.9)	6.02 (2.064-17.55)	0.004*
	Prisoners	12 (2.2)	5 (17.9)	9.66 (3.14-29.70)	0.001*
5	Comorbidities				
	Diabetes	18 (3.3)	0 (0)	n/a	0.99
	Malignancy	11 (2)	1 (3.6)	1.73 (0.266-11.72)	0.46
	Insufficient renal function or on dialysis	5 (0.9)	0 (0)	n/a	0.99
	Organ transplantation	2 (0.4)	0 (0)	n/a	0.99

Information: *significant (p-value < 0.05); n/a: not applicable due to small number of event; **missing value: Country of birth 2 (0.03%), Previously diagnosed TB 59 (10.3%), previous LTBI treatment 67 (11.7%); ‡Urban domicile : Amsterdam, Rotterdam, The Hague and Utrecht; TB, tuberculosis; ETB, extra-pulmonary tuberculosis; PTB, pulmonary tuberculosis; MDR-TB, multidrug-resistant tuberculosis; XDR-TB, extensively drug-resistant tuberculosis; LTBI, latent tuberculosis infection; CI, confidence interval.

Table S3 . Poor outcome of TB treatment between males and females among MDR-TB patients (N= 103)

No	Characteristics	Male (n=53)			Female (n=50)		
		Poor outcome		p-value	Poor outcome		p-value
		No (n=42)	Yes (n=11)		No (n=49)	Yes (n=1)	
1	**Socio-demographic**						
	Age (years):			0.82			1.00
	18-24	10 (23.8)	2 (18.2)		16 (32.7)	0 (0)	
	25-64	30 (71.4)	8 (72.7)		33 (67.3)	1 (100)	
	65+	2 (4.8)	1 (9.1)		0 (0)	0 (0)	
	Foreign-born patients	40 (95.2)	11 (100)	1.00	49 (100)	1 (100)	n/a
	Urban domicile	9 (21.4)	5 (45.5)	0.13	12 (24.5)	0 (0)	1.00
2	**Current TB diagnosis**						
	Pulmonary diagnosis:			0.68			0.66
	ETB	13 (31)	2 (18.2)		8 (16.3)	0 (0)	
	PTB	23 (54.8)	7 (63.6)		32 (65.3)	1 (100)	
	ETB + PTB	6 (14.3)	2 (18.2)		9 (18.4)	0 (0)	
	Type of TB location:			0.96			1.00
	Lungs	26 (61.9)	7 (63.6)		39 (79.6)	1 (100)	
	Miliary and central nervous system	3 (7.1)	1 (9.1)		0 (0)	0 (0)	
	Others	13 (31)	3 (27.3)		10 (20.4)	0 (0)	
	Diagnosed by a doctor abroad	2 (4.8)	0 (0)	1.00	3 (6.1)	0 (0)	1.00
3	**History of TB disease & treatment**						
	Previously diagnosed with TB*	9 (23.7)	3 (27.3)	1.00	9 (21.4)	1 (100)	0.23
	Previously LTBI treatment**	3 (8.1)	0 (0)	1.00	2 (4.9)	0 (0)	n/a
4	**The risk group of TB**						
	TB contacts	1 (2.4)	1 (9.1)	0.38	3 (6.1)	0 (0)	1.00
	Immigrants	7 (16.7)	1 (9.1)	1.00	13 (26.5)	0 (0)	1.00
	Asylum seekers	12 (28.6)	4 (36.4)	0.72	15 (30.6)	0 (0)	1.00

	Illegal residence persons	5 (11.9)	0 (00	0.57	0 (0)	0 (0)	n/a
	Homeless individual	2 (4.8%)	2 (18.2)	0.19	0 (0)	0 (0)	n/a
	Alcohol dependence	0 (0)	1 (9.1)	0.21	0 (0)	0 (0)	n/a
	Substance abuse	2 (4.8)	3 (27.3)	**0.05**	0 (0)	0 (0)	n/a
	Health care workers	1 (2.4)	0 (0)	1.00	0 (0)	0 (0)	n/a
	Travellers from/in endemic areas for more than 3 month	2 (4.8)	0 (0)	1.00	3 (6.1)	0 (0)	1.00
	Prisoners	4 (9.5)	0 (0)	0.57	0 (0)	0 (0)	n/a
5	Comorbidities						
	Diabetes	2 (4.8)	1 (9.1)	0.51	1 (2)	0 (0)	1.00
	Malignancy	2 (4.8)	0 (0)	1.00	0 (0)	0 (0)	n/a
	Insufficient renal function or undergoing dialysis	0 (0)	0 (0)	n/a	0 (0)	0 (0)	n/a
	Organ transplantation	0 (0)	0 (0)	n/a	0 (0)	0 (0)	n/a

Information. n/a : not applicable due to no case in the comparator; * missing data: male (4) & female (7); ** missing data : male (5) & female (9);‡ Substance abuse in male: OR 7.5 (95%CI 1.074-52.28).
